# The barrier and beyond: Roles of intestinal mucus and mucin-type O-glycosylation in resistance and tolerance defense strategies guiding host-microbe symbiosis

**DOI:** 10.1080/19490976.2022.2052699

**Published:** 2022-04-05

**Authors:** Kirk Bergstrom, Lijun Xia

**Affiliations:** a Department of Biology, University of British Columbia, Okanagan Campus, 3333 University Way, Kelowna, British Columbia V1V 1V7, Canada; b Cardiovascular Biology Research Program, Oklahoma Medical Research Foundation, OK, Oklahoma 73104, USA; c Department of Biochemistry and Molecular Biology, University of Oklahoma Health Sciences Center, OK, Oklahoma 73104, USA

**Keywords:** Mucin-type O-glycans, mucus, microbiota, disease tolerance, host-defense, colon

## Abstract

Over the past two decades, our appreciation of the gut mucus has moved from a static lubricant to a dynamic and essential component of the gut ecosystem that not only mediates the interface between host tissues and vast microbiota, but regulates how this ecosystem functions to promote mutualistic symbioses and protect from microbe-driven diseases. By delving into the complex chemistry and biology of the mucus, combined with innovative in vivo and ex vivo approaches, recent studies have revealed novel insights into the formation and function of the mucus system, the O-glycans that make up this system, and how they mediate two major host-defense strategies – resistance and tolerance – to reduce damage caused by indigenous microbes and opportunistic pathogens. This current review summarizes these findings by highlighting the emerging roles of mucus and mucin-type O-glycans in influencing host and microbial physiology with an emphasis on host defense strategies against bacteria in the gastrointestinal tract.

## Introduction

### If you can’t beat ’em, join ’em

Host defense is an integral part of living with our gut microbiota. Whether the symbiotic microbiota represent resident commensals, mutualists, or overt parasites (pathogens), host innate defense strategies are in place to defend against – or regulate interactions with – all classes of symbionts with the same end goal: to prevent disease, and, ideally, promote a mutualism to enhance fitness of host and symbiont. Two fundamental ways exist in which to achieve this goal: First, we can “resist” the pathogen by regulating numbers via preventing colonization or through direct killing mechanisms.^1,2^ Resistance strategies are well characterized, i.e., colonization resistance, production of antimicrobials that lyse target cells, opsonization with complement and antibodies, and phagocytosis. By reducing pathogen burdens, we reduce the stimulus and number of virulence factors and toxins that cause damage and disease and thereby enhance host fitness. However, resistance comes at a cost – an ongoing evolutionary arms race and selection pressures as both host and microbe fight for survival over generations.^2,3^ Further, tissues such as the lung, skin, and reproductive and digestive tracts are constantly exposed to the environment: The digestive tract is an extreme example where >10 trillion bacteria alone exist, making resistance effectively futile. This necessitates the second strategy: promoting “tolerance” to the microbe (or microbially-derived toxin) so as to prevent its ability to cause tissue damage and reduced host fitness.^1,4^ Indeed, tolerance strategies are required and employed that not only preserve the gut microbiota, but can ultimately enhance their fitness in ways that enhance our own.^1^ Such strategies include dampening host inflammatory potential, for example, through IL-10^1^ or other negative regulators of inflammation, which maintains a stable microbiota rich in alpha diversity^5^ that promotes colonization resistance against pathogens,^6^ and increases our energy extraction from the diet.^7^ Importantly, these strategies are not always clear-cut: Situations arise where a defense strategy is an asset in one context (promoting resistance or tolerance), but a liability in another, by promoting infection and disease. One major feature of host innate defenses that has emerged to promote resistance and tolerance to the microbes – and sometimes disease susceptibility – is the heavily O-glycosylated mucus system. A number of recent reviews have highlighted the importance of how mucin-type O-glycans contribute to homeostasis.^8–10^ However, the broader significance of the relationship between host glycans, host–microbe interactions, and host defense is still poorly defined, especially at the interface where host and microbe interact, which largely dictates the physiologic outcome – and evolutionary trajectories – of both host and microbe. This review focuses on how intestinal secretory mucins, by virtue of their O-glycone, specifically contribute to host defense strategies, with an emphasis on emerging tolerance defense mechanisms against the bacterial symbiotic microbiota.

## Gut mucus and mucin-type O-glycans

Gut mucus is comprised of a polymeric network of the gel-forming glycoprotein MUC2, which belongs to the mucin (MUC) family.^9^ The family comprises approximately 20 members in humans, and can be broadly divided into membrane-bound (MUC1, MUC3A, MUC3B, MUC4, MUC12, MUC13, MUC15, MUC16, MUC17, MUC21, and MUC22); gel-forming secreted (MUC2, MUC5AC, MUC5B, and MUC6); and non-gel-forming-secreted (e.g., MUC7, MUC8, MUC9, and MUC20) forms.^9,11^ These mucins collectively contribute to regulating host–environment interactions at mucosal surfaces through myriad mechanisms ranging from forming physiochemical barriers to regulating signal transduction pathways in epithelial cells.^9,12^ In the colons of both mice and humans, MUC2 (Muc2 in mice) is the major gel-forming mucin comprising the mucus system. This is produced exclusively by goblet cells of the intestinal and colonic epithelium^13^ where monomers of MUC2 are glycosylated within the proline, serine, threonine-rich (PTS) domains (discussed below) and dimerize at the C-terminus, and form trimers at the N-termini.^14–16^ The resulting trimer dimers then covalently bind one another to form enormous polymers that are densely packaged in secretory granules within goblet cells theca and released in a constitutive or stimulated fashion.^17^ Once released, they are hydrated and expand to form the gel that can function as a barrier (discussed below), and lubricant to facilitate passage of luminal contents without damaging tissues, and contribute to the overall function of the intestinal ecosystem (discussed below). Most of these vital functions of MUC2 are governed by its O-glycone (i.e., O-linked glycan repertoire).

**Figure 1.**
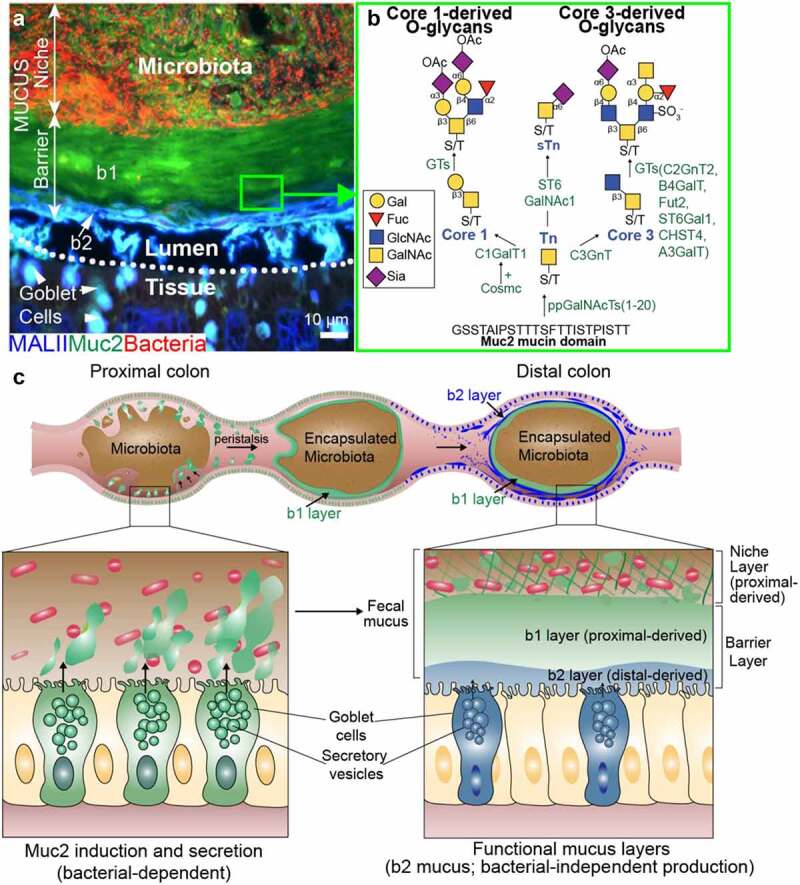
**A**. Structure of the mucus layer in the mouse colon. Representative cross section of a carnoy’s fixed paraffin-embedded wild-type mouse distal colon with fecal pellet intact. The tissue was triply labeled with antibody for Muc2 (green), the Mal-II lectin, (blue), and bacteria in red (EUB338 FISH probe), reproduced from ref. [47]. b1 = proximal colon derived barrier layer; b2 = distal colon derived barrier layer. **B**. Biosynthetic pathways of complex glycans on Muc2. Details in text. **C**. Diagram of mucus formation. Type 1 mucus is produced by the proximal colon goblet cells in a microbiota-dependent fashion and gives rise to niche and b1 barrier layer. Type 2 mucus (sulfated, MALII+) is produced by distal colon goblet cells and gives rise to b2 barrier layer, independent of the microbiota.

MUC2 is comprised mainly of glycans, >80% by weight, which are made up of >100 structures containing combinations of at least five monosaccharides: galactose (Gal), N-acetylgalactosamine (GalNAc), N-acetylglucosamine (GlcNAc), sialic acid (Sia), and Fucose (Fuc).^18,19^ The main type of glycosylation on MUC2 is “O-linked”, due to modification of – OH (hydroxyl) groups of serine/threonine (Ser/Thr) abundant in the MUC2 PTS domain with GalNAc and subsequent extension by many glycosyltransferases (Figure 1B).^18^ The initiating structure GalNAcα-O-Ser/Thr (a.k.a. “Tn antigen”) is the building block for complex glycans, and is found mainly on mucins or mucin-like glycoproteins, hence called mucin-type O-glycosylation (O-GalNAc glycosylation, O-glycosylation hereafter).^18–20^ The biosynthesis of Tn antigen is controlled by a family of polypeptide *N*-acetylgalactosaminyl transferases (ppGalNAcT), which constitutes 20 isoenzymes in humans.^21^ The Tn structure, which normally is not an exposed structure in most tissues, is further modified by at least two major glycosyltransferases: core 1 β1,3 galactosyltransferase (C1GalT1) and core 3 β1,3 N-acetylglucosaminyltransferase C3GnT (also called β3GnT6).^18,22^ C1GalT1 adds a Gal residue in β1,3 linkage to Tn, forming the core 1 structure (Galβ1-3GalNAcα-O-Ser/Thr), whereas C3GnT adds GlcNAc in β1,3 linkage to Tn to form the core 3 structure GlcNAcβ1-3GalNAcα-O-Ser/Thr (Figure 1b).^18,22^ In some pathologic states, ST6GalNAc1 can modify Tn with Sia, forming the cancer carbohydrate sialyl-Tn (sTn)^23^ (Figure 1B). Importantly, C1GalT1 requires core-1 GalT1-specific molecular chaperone (Cosmc), encoded by the X chromosome-linked *C1GALT1C1* gene^24^ for its activity. Core 1 or 3 structures can then be branched by members of the core 2 β1-6 N-acetylglucosaminyltransferase (C2GnT) family to form core 2 and 4 structures, respectively, and subsequently further extended and branched by concerted actions of diverse families of glycosyltransferases including sialyltransferases, fucosyltransferases, and other Gal/GalNAc and GlcNAc transferases, as well as glycan modifying enzymes, such as sulfotransferases and O-acetyltransferases.^23^ The resulting oligosaccharides exhibit monosaccharides linked in various combinations, lengths, and anomeric configurations to ultimately form a heterogeneous three-dimensional display to the microbiota^25^ (Figure 1B).

The glycan structures are often capped with fucose or Sia, and will frequently form important antigenic structures including the ABO blood group determinants, Sd^a^/Cad antigen, and Lewis structures, which are found on other glycoconjugates in the body.^23^ The great diversity of glycan structures, comprised of at least >100 unique structures as revealed through advanced mass spectrometry-based approaches,^26–28^ have long implied important biologic functions that have been traditionally challenging to define due to the complexity of glycosylation. We and others have focused in vivo studies in mice to provide definitive data on the expression and function of mucin-type O-glycans in the intestinal tract. These studies have revealed central roles of mucin-type O-glycans in innate host defense strategies that protect from inflammatory interactions with our commensal microbiota and infection-induced damage from enteric pathogens.

## Roles of mucus and their glycans in tolerance defense mechanisms

### Mucus barrier function as a tolerance-defense mechanism

#### MUC2 and the mucus barrier

An established function of the gut mucus system is its role in barrier function in the gut toward the microbiota. The mucus is actually a multi-layered gel with two major structures (Figure 1A): i.) A barrier layer that is mostly impenetrable to the microbiota leading to their spatial segregation from the distal colon mucosa,^29^ and ii.) A niche layer that many microbes readily colonize and thrive in.^30^ Support for the existence of these mucus structures comes from over two decades of research in humans and mammalian model systems including mice and rats. The gold standard to visualizing this layer comes from fixing colonic tissues in anhydrous Carnoy’s solution, which preserves the mucus structure in standard paraffin-embedded colonic sections, which is visualized upon staining with histochemical approaches that label glycoconjugates (periodic acid-Schiff (PAS) for neutral and acidic mucins, and Alcian blue for acidic mucins), or epifluorescent labeling with antibodies to Muc2 or lectins targeting glycan epitopes on MUC2.^31^ The majority of the characterization has been done in rodents, with limited studies in humans. The mucus has also been observed in native settings by directly staining mucosal tissue with PAS.^32^ An independent technique to quantify native mucus via micro pipetting on explants in buffer systems^33,34^ has supported the notion for “loose” (i.e., easily removed) and “firm” (tissue-adherent) mucus layers that vary in thickness along the intestinal tract, being thickest within the colon.^33^ Svojall and Hansson and colleagues have developed an elegant explant system in which the native mucus can be seen and interrogated using bacteria or bacteria-sized beads and confocal and quantitative imaging approaches.^34^ Both human and mouse explants can be studied in this system, each showing a similar conserved function of the native mucus in both species.^34^ Collectively, these studies have led to a classic model in which distal colon goblet cells secrete mucus on their surface, which expands to form a dense adherent inner layer that gives rise to a loosely associated outer layer that is easily removed.^9,33,35^ This model has served as a vital starting point in our rapidly evolving understanding of this complex system.

This structure and function of Muc2 was confirmed using mice genetically deficient in Muc2 (*Muc2^−/−^*),^35^ originally developed by Velchich et al.^36^ Consequently, *Muc2^−/−^* mice have bacteria invading distal colon crypts that are normally sterile,^35^ and develop spontaneous colitis,^37^ colorectal cancer,^36^ and are susceptible to enteric infections (discussed more below).^11,38–40^ Similarly, *Winnie* mice, which are defective in their ability to produce normal levels of Muc2 due to a point mutation induced by N-ethyl-N-nitrosourea (ENU), develop spontaneous colitis, although endoplasmic reticulum stress due to Muc2 misfolding is implicated in the pathogenesis.^41^ These studies are consistent with phenotypes observed in the adult and pediatric inflammatory bowel disease (IBD).^42–44^ Because of the exceptional density and diversity of the colon microbiota with ~40–300 billion bacteria alone/gram of feces,^45^ comprising 300–500 species,^46^ this barrier function of mucus can be considered an important form of tolerance to the microbiota, allowing our co-existence – often mutualistic – despite the potential of the microbes to cause harm,^46^ for example, in sepsis or IBDs such as ulcerative colitis (UC) and Crohn’s disease (CD).

#### New insights into mucus barrier functions

Recently, we have demonstrated that O-glycosylated mucus is more complex and intimately associated with the microbiota than previously known.^47^ We found the mucus barrier layer encapsulates discrete portions of the microbiota community within fecal pellets, a finding also observed by Kamphuis et al.,^48^ and is actually comprised of two distinct mucus subtypes, one which forms a major barrier layer, designated the “b1” layer derived from the proximal colon goblet cells, and a newly discovered “b2” layer, a minor barrier layer derived from the distal colon goblet cells.^47^ These can be distinguished by labeling with the lectin *Maackia amurensis* lectin (MAL)-II which bound to a sulfated glycan in the b2 minor layer^47^ (Figure 1a–c). The type 1 mucus making up the b1 layer also made up the niche layer the microbes reside in; however, the type 2 (MALII^+^) mucus making up the b2 layer does not frequently interact with the microbiota.^47^ The absence of the b1 layer was sufficient to desegregate the microbiota from the tissues and induce spontaneous colitis; however, both spontaneous and DSS-induced colitis were worse when both b1 and b2 layers were compromised.^47^ Thus, both proximal and distal colon mucus work to promote barrier function and tolerance to the microbiota.^47,49^ This new understanding of the fecal-associated rather than tissue-associated mucus barrier led to our redefinition of the mucus system that now describes a microbe-centric “niche” layer and “barrier” layer, the latter divided into the b1 and b2 sublayers (Figure 1).

Critically, the phenomena of the fecal mucus barrier association is conserved in other species including rat (which also has the b1/b2 layers), baboon,^47^ and human,^47,50^ although the b1/b2 subtypes have not been confirmed in the latter. Still, there is thick and clear mucus on the periphery of well-formed human and baboon stool that is impenetrable to the microbiota.^47^ The similarities between the rodent and primate/human mucus suggest a similar proximal colon-mediated mechanism leads to association of fecal mucus in humans and raises important questions as to what leads to mucus defects in human diseases like UC. Further, these studies raise new questions on the relative contribution of mucus production from proximal and distal sites to the niche and barrier functions of mucus. For example, are the native mucus studies in the mouse explant model system using distal colon representing the type II sulfated mucus? Does the encapsulating mucus on human feces represent a different type compared to that present in distal colon goblet cells as observed in rodents?

The newly discovered b2 mucus barrier and the different goblet cell populations are consistent with recent studies identifying novel goblet cell subsets by single-cell transcriptomics. Using RedMUC2-Tg mice, which express a human MUC2 fused to mCherry, and originally developed by Hansson and colleagues,^51^ Nystrom et al. showed an intercrypt goblet cell subset in the distal colon that promotes mucus barrier formation.^52^ These are likely contributing mainly to the b2 layer. Further, the sentinel goblet cells (SenGCs) discovered by Birchenough et al.^51^ rapidly respond to bacteria penetrating the mucus barrier through a toll-like receptor (TLR) and NACHT, LRR (leucine-rich repeat), and PYD (pyrin domain) domain-containing 6 (NLRP6)-driven secretory response.^51^ The role of O-glycans in this setting is unclear, but are likely promoting the stability of the mucus in these contexts.^53^

#### When mucus barrier function is dispensable for tolerance

As critical as the mucus barrier is for tolerance to the microbiota, the absence of segregation is not always inherently inflammatory. The proximal colon and cecum do not have a robust mucus barrier layer and microbes are frequently associated with tissues.^47,54^*Bacteroides* species inhabit the proximal colon crypts as a dedicated niche.^54^ Thinner mucus is still functional and does not directly lead to loss of tolerance defense toward the microbiota as evidenced by lack of overt idiopathic inflamamation.^55^ Polyethyleneglycol treatment disrupts the mucus, but does not itself instigate inflammation, although it is associated with altered immunoglobulin levels and a lasting impact on the microbiome.^56^ The cecum has similar microbial load as the colon, but very little signs of inflammation towards the microbiota, despite the loss of Muc2.^38^ However, loss of Muc2 renders cecum more susceptible to *C. rodentium* infection by limiting colonization, showing that Muc2 is important in host defense by regulating colonization likely independent of its barrier roles.^38^ These studies suggest there are contexts when Muc2 is dispensable for tolerance to the microbiota, which is driven instead by other poorly defined mechanisms, yet still protective for host defense against overt pathogens independent of its barrier roles. Defining when mucus is necessary for mutualistic symbiosis vs. not is important for our complete understanding of the roles of mucins in health and disease.

## O-glycans and mucus barrier-dependent tolerance mechanisms

### Core 1-derived O-glycans and mucus barrier functions

In vivo studies have revealed an interesting interplay exists between the core 1- and core 3-dependent complex O-glycans in mice and humans. C1GalT1 is ubiquitously expressed.^57,58^ In mice, core 1-derived O-glycans dominate, making up >90% of the total complex O-glycans.^27^ Mice conditionally lacking core 1-derived O- glycans in gut epithelium (IEC *C1galt1^−/−^*) show truncation of O-glycans down to Tn mainly in small intestinal and distal colon epithelium.^59^ IEC *C1galt1^−/−^* mice develop spontaneous microbiota-dependent colitis, associated with loss of mucus barrier integrity and microbial invasion.^59^ Consistent with this, Cummings and colleagues^60^ using mice conditionally lacking *Cosmc* in gut epithelium similarly developed microbiota-dependent colitis through impacts on mucus integrity.^60^ Interestingly, the *Cosmc* system provides an important model to study the impact of X-linked inactivation and glycosylation since heterozygous females (*Cosmc^−/x^*) show a mosaic pattern of glycan truncation.^60^ These two independent model systems underscore the importance of core 1 O-glycosylation to tolerance defense against the microbiota.

### Core 3-derived O-glycans and mucus barrier functions

**Figure 2.**
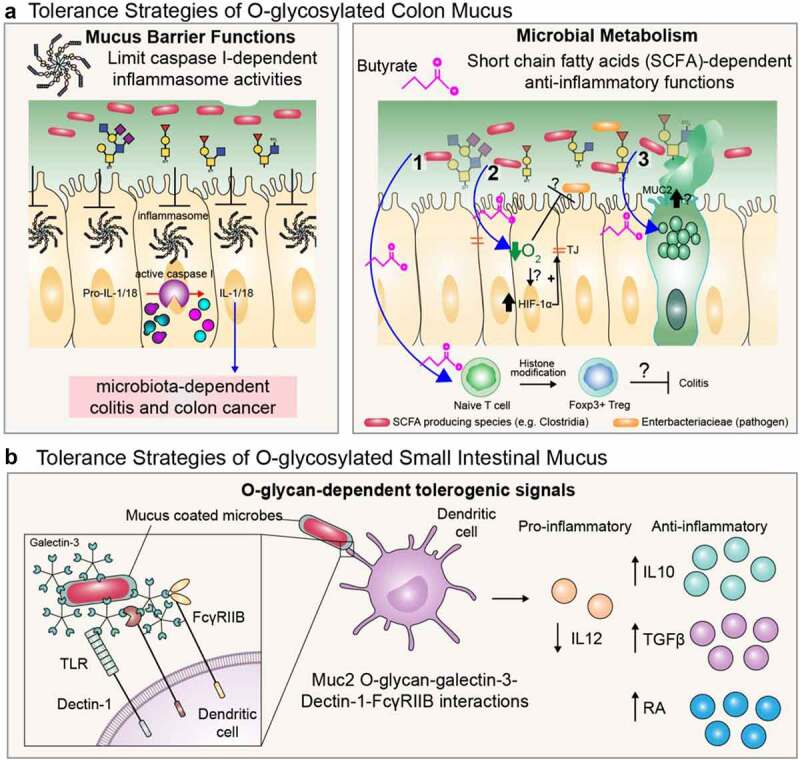
Examples of collective actions of O-glycans promoting tolerance defense strategies of mucus. A. tolerance strategies of colon mucus. *left panel*; mucin-type O-glycan-dependent barrier functions limit hyperactive microbiota-dependent inflammasome activities that drive chronic colitis and colitis-associated cancer. *right panel*; microbial metabolism of O-glycans leads to anti-inflammatory metabolite production by mucin-utilizers. the pathways shown reflect established functions of SCFA such as butyrate, including 1.) inducing differentiation of Tregs; 2.) repleting epithelial O_2_ via oxidation of butyrate which impairs pathogenic enterobacteriaceae family colonization and promotes HIF-1α-dependent barrier function (refs.113,114); and 3.) inducing MUC2 expression within goblet cells (refs 117, 118). the question marks refer to whether or not SCFA produced by commensal-dependent metabolism of O-glycans on mucus occur at levels that can drive a similar tolerogenic response. **B**. tolerance strategies of small intestinal O-glycosylated mucus. mucus coats bacteria to promote an anti-inflammatory gene expression program in dendritic cells.

In contrast to core 1 dominance in mice, core 3-derived O-glycans dominate the human colon.^27,28^ Nevertheless, gene-targeted strategies have shown core 3 O-glycans in mice still have important physiologic roles. Consistent with the minor contribution of core 3-derived O-glycans to homeostasis at baseline, mice lacking core 3-derived O-glycans (*C3GnT^−/−^)* do not develop overt spontaneous disease but are highly susceptible to acute injury by dextran sodium sulfate (DSS) and colorectal cancer induced by DSS-Azoxymethane.^22^ This is associated with impaired mucus barrier function.^22^ This lesser impact is most likely attributed to the major activities of C1GalT1/Cosmc-dependent glycosylation. We have demonstrated that C3GnT is uniquely expressed in a subset (~50%) of goblet cells in the proximal large intestine (cecum and proximal colon) of mice.^53^ Core 3-derived O-glycans alone can, remarkably, still can retain barrier activities of the encapsulating mouse mucus,^53,61^ although this may depend on the relative mucin-degrading activities of the specific microbial consortium since studies in the IEC *C1galt1^−/−^* mice show both intact or degraded mucus.^53,59,62^ Interestingly, Hansson and colleagues have shown that the glycosylation profile of remaining core 3 O-glycans in IEC *C1galt1^−/−^* mice is distinct from complex core 1-derived O-glycans, with core 3-derived structures showing a higher degree of glycan extension, sialylation, and sulfation vs. core 1.^27^ Core 3-derived structures in mice vs. humans is also different,^27^ likely due to the context of glycosyltransferase families between species, although this has yet to be determined. The importance of core 3 is highlighted in the generation of compound mutant IEC *C1galt1^−/−^; C3GnT^−/−^* (DKO) which show a rapid disintegration of the encapsulating mucus and accelerated onset of colitis after weaning, associated with a complete truncation of all O-glycans throughout the colon.^53^ These mice revealed the important principle that the degree of O-glycosylation impacts mucus functions and colitis potential. Importantly, DKO mice also developed spontaneous colorectal cancer.^63^ Mechanistically, this was associated with chronic hyperactivation of caspase 1-dependent epithelial inflammasomes by the microbiota. This in turn led to a tumor-promoting microenvironment characterized by increased DNA-damaging reactive nitrogen species by neutrophils, and proliferation-inducing inflammatory cytokines (IL-6, −17), which together drove inflammation-dependent cellular transformation and tumorigenesis.^63^ Critically, ablation of inflammasome activities lead to near complete resolution of inflammation and tumors despite microbial desegregation.^63^ These studies reveal that core 1- and 3- O-glycans are critical to promote tolerance to the microbiota by stabilizing the mucus barrier to segregate the microbiota and to prevent their ability to cause cancer-inducing inflammasome activation in epithelial cells (Figure 2A).

### Dietary influences on O-glycan-dependent barrier functions

Mucins and microbiota are typically in a context of varied diets. The overall glycosylation profiles seem relatively stable in the face of many diets; however, there are limited studies to suggest diet directly impacts glycosylation. Prominent members of the microbiota, including *Bacteroides spp, Akkermansia muciniphila, Ruminococcus spp*, and others can employ a variety of Carbohydrate-Active enZymes (CAZymes) including exoglycosidases,^64,65^ and the more recently described endoglycosidases^66^ to degrade O-glycans on Muc2. Aggressive mucus-degrading communities can develop in response to consuming fiber-free or Western-style diets, which force foraging on host glycans leading to thinning of the mucus,^67,68^ suggesting the host is not able to replace the complex glycans at the rate they are consumed. However, these mucin-degrading communities are unable to completely degrade the mucus barrier to instigate inflammation, showing the power of Muc2 O-glycans in protecting against aggressive communities in these extreme settings. In support of this notion, we have provided evidence that non-optimal glycosylation renders the mucus more easily degraded by the microbiota on polysaccharide-deficient diets.^47^ Still, these thinner mucus layers are associated with increased susceptibly to overt pathogens including *Citrobacter rodentium*.^67^

Collectively, these results indicate that O-glycans are required for the barrier functions of mucus that overall promote tolerance to the microbiota by stabilizing the encapsulated microbiota and preventing microbiota-dependent activation of epithelial inflammasomes that drive chronic tumor-promoting inflammation. However, it remains to be determined whether specific O-glycans on mucus (e.g., fucosylated, sialylated) mediate this protection and whether the tolerance effect is purely a matter of segregation of the microbiota or some aspect of microbial modulation (compositional or functional). Indeed, as described below, recent studies are pointing to roles of glycans in modulating microbial community structure and function.

## O-glycans in relation to MUC2-dependent tolerance-mediated defense along the intestinal tract

### Relationship of regiovariation of O-glycosylation to microbial disease along the intestinal tract

Both humans and rodents show regiospecific O-glycosylation within the intestine. Using electrospray ionization quadrupole time-of-flight tandem MS, Robbe et al. (2004)^28^ have shown mucin-type O-glycans extracted from crude mucins scraped from different regions of the human intestinal tract were mainly core 3/4-based, but also built on core 2 in the colon. Core 5 (GalNAcα1-3GalNAcα-O-Ser/Thr) structures were also present throughout the intestinal tract. There were clear regional variations in terminal structures carrying blood group determinants: core 4-based fucosylated structures dominated the small intestine, including histo-blood group antigens A and H on the terminal positions.^28^ In the colon, Sd^a^/Cad epitopes were more common, with the distal colon being most abundant with this epitope, along with core 2-based sulfoLe^x^ and higher overall acidic glycans.^28^ Notably, these studies were from individuals of blood type A, Le^b^, which may influence the glycosylation patterns. In mice, while glycosylation profiles are different than humans,^27^ the regiovariation is conserved: Using LC/MS on extracted and gel-purified murine mucus, it was shown that the small intestine was dominated by core-2 type sialylated and sulfated complex O-glycans but relatively low fucosylated structures (in contrast to human), while the colon exhibited markedly higher level of fucosylated structures as well as increased levels of sialylated O-glycans in the proximal colon, and – similar to humans – a higher level of sulfated glycans in the distal colon.^69^ The common theme of higher sulfated glycans in the distal colon of both species is notable as this region is uniquely susceptible to microbiota-dependent colitis in both human UC and mouse colitis models.^59,70^ There are few studies that have investigated the relationship of these different O-glycan profiles to host-microbiota mutualism, but have long been suggested to dictate microbial attachment sites^71^ and may be regulated by select members of the community (discussed below). Importantly, we have shown the role of mucin-type O-glycosylation in host-bacterial homeostasis appears to be more critical in the colon vs. the small intestine. Mice with loss of core-1 derived O-glycans in the gastric epithelium develop gastritis and gastric cancer independent of microbes, such as *Helicobacter* and other microbes, as shown by antibiotic treatment.^72^ Loss of small intestinal O-glycans also predisposes mice to spontaneous duodenal tumors linked to microbiota-independent low-grade inflammation.^73^ In contrast, O-glycosylation is essential to protect from microbiota-dependent inflammation in the colon as mentioned above. These studies are not surprising in light of the known gradient in density and diversity of the microbiota along the intestinal tract, with microbial loads being up to 5 orders of magnitude higher in the colon vs. the small intestine.^46^ However, they do point to novel tolerance functions of glycans to non-microbial derived damaging agents.

### It takes two (trillion) to tango: mutual influences between microbes and glycans that promote tolerance-mediated host defense

There is an intimate relationship between mucin-type O-glycans and the microbiota along the GI tract signifying a mutually influential interaction. Several studies with germ-free and gnotobiotic mice have reported microbial influences on mucus expression and its O-glycosylation that point to tolerance strategies. The microbiota is known to be required for complete formation of the mucus system, which requires seven weeks to reach a steady state.^74^ We have shown the microbiota specifically induces Muc2 expression and secretion in proximal colon goblet cells, which leads to their own encapsulation.^47^ The influence of the microbiota on the mucus is consistent with studies describing microbiota-dependent mucus thickness;^55^ however, whether these findings reflect microbial influences of proximal goblet cell function is not clear. The specific induction in proximal colon goblet cells was necessary to prevent spontaneous and acute distal colon colitis,^47^ which is known to be microbiota-dependent.^53^ Therefore, microbe-dependent mucus production in the proximal colon can be argued to be an essential tolerance defense strategy toward the dense microbiota. The secretion mechanisms remain ill-defined but are independent of canonical and non-canonical inflammasome signaling,^74^ consistent with explant studies in the distal colon by Birchenough and colleagues.^75^ Future studies will need to address its role against overt pathogens. Based on studies in *Muc2^−/−^* mice with pathogens including *C. rodentium*, it can be inferred that loss of this encapsulation leads to pathogenic microcolonies on the ulcerative mucosal surface and uncontrolled bacterial dissemination and sepsis-like conditions in these animals.^38^

Microbes also influence O-glycosylation patterns within the mucus. Early studies by Hooper and Gordon have shown *B. theta* monocolonization can secrete a factor that induces expression of fucosylated glycans in the small intestine, which it forages as a nutrient source.^76^ Consistent with this, using lectin profiling of tissues, Freitas et al. (2005) have demonstrated monocolonization with *Bacteroides* or its spent media drastically changes lectin staining of goblet cells that indicate changes in fucosylation, as well as other goblet cell glycosylation pathways in small intestine and colon.^77^ More recently, Hansson and colleagues used mucins purified from mucosal scrapings to explore this question on a biochemical level, showing by parallel proteomics and glycomics via LC/MS in germ-free vs. ex-germfree mice that microbes induce changes in O-glycan structure and expression of pertinent glycosyltransferases and glycan-modifying enzymes.^78^ Whether these changes are induced in specific goblet cell subsets or with the encapsulating mucus would be informative. Interestingly, lectin staining patterns using the α1,2 fucose-targeting UEA1, show a diffuse staining pattern of this glycan in germ-free conditions throughout the fecal pellet that redistributes to the periphery, where the mucus, is upon conventionalization, likely reflecting metabolism by the microbiota.^47^ It is also noteworthy which types of O-glycans are independent of the microbiota. For example, it is striking that the distal colon mucus phenotype (MALII^+^) and the proximal colon lectin staining profiles was independent not only of diverse genetic backgrounds of mice, but also the microbiota, as the b2 layer and its source (the MALII+ goblet cells) were intact independent of microbiota status.^47^ How each of these changes reflects a host defense strategy is still unclear, but studies point to direct impacts on community structure and function.

### O-glycans driving community eubiosis as a potential tolerance strategy

As the microbiota can shape its own mucin environment^55,74^ including its encapsulation and glycosylation,^47,77,78^ it is expected this would also translate into an impact on community composition. To this end, Muc2 has recently been shown to modulate microbiota communities in ways that promote eubiosis, that is, a commensal or mutualistic symbiosis.^79^ Chadee and colleagues have shown it can suppress development of a colitogenic microbiota.^79^ We have shown core 1- and 3-derived O-glycan-dependent encapsulation of the microbiota influences established mucin utilizers including *Akkermansia muciniphila* and several *Bacteroides* species, which was linked to the proximal colon-dependent bacterial replication rates.^47^ Consistent with this, Bäckhead and colleagues have demonstrated loss of core 1-derived O-glycans increases Bacteroidetes at the expense of Firmicutes, which was associated with increased susceptibly to acute gut injury.^80^ Using a similar model system with a more detailed immunologic and bioinformatic approach, Braun and colleagues have also shown gut epithelial core 1 deficiency leads to fluxes in regulatory cell (T_reg_) production and colitis severity in the mucosa within the first 12 weeks of life, with concomitant defective core 1 O-glycan-dependent increases in the Clostridiales order associated with proinflammatory RORgt^+^CD4^+^T cells populations and an altered mucosal and luminal metabolome.^81^ Notably, this study also linked polymorphisms in *C1GALT1* to an altered microbiota in Crohn’s’ disease patients.^81^ Deficiency in the X-linked *Cosmc* gene (*C1GALTC1*), essential for core 1 O-glycan synthesis, was also shown by this and an independent study^59^ to be linked to IBD. Consistent with this, Cummings and colleagues have shown *Cosmc* loss leads to sex-specific impacts on mucosal-associated communities, including a reduction and increase in *Bacteroides* and *Helicobacter* genera, respectively.^60^ Whether these changes reflect a more inflammatory community is not clear, as core 1- O-glycan-dependent microbial shifts did not necessarily translate to a more inflammatory population, perhaps due to remaining core 3/4 type O-glycans in these models.^61^ However, Sonnenburg and colleagues have recently shown, by supplementing the diet with O-glycan-like human milk oligosaccharides (HMOs) or mucin-type O-glycans purified from commercially derived porcine gastric mucin, functionally dysbiotic (antibiotic or disease-associated) communities can be shifted back to a eubiotic (healthy-associated) state.^82^ While this has implications for mucin-based therapies, whether this is enhanced by native mucin (i.e., glycans bound to peptide core) or using actual human mucin is unclear. That said, these studies suggest that complex O-glycans contribute to overall tolerance to the microbiota by shaping community composition and function. While these “corporate” functions of O-glycans are clearly protective, evidence also suggests specific glycan subsets contribute to microbial tolerance.

### Role of fucosylation in tolerance-defense strategies

**Figure 3.**
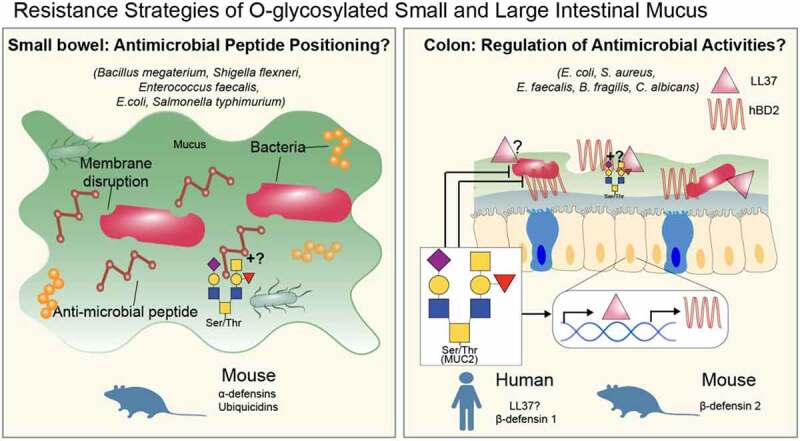
Examples of collective actions of O-glycans promoting resistance defense strategies of mucus. Both small and large intestinal mucus can be a reservoir for antimicrobial peptides (AMPs) that can directly kill pathogenic microbes. For the small intestine (*left panel*): the question mark refers to whether O-glycans play direct roles in binding and positioning antimicrobial peptides or play indirect roles in AMP positioning by promoting mucus stability. For the colon (*right panel*), mucus O-glycans can stimulate antimicrobial gene expression, while paradoxically inhibiting hBD2-dependent killing of bacteria. The question marks refers to whether mucin O-glycans can similarly modulate LL37 killing capacity and binding of these antimicrobials to colon mucus. The role of b1 and b2 mucus in this process also unknown.

Fucosylation impacts microbial metabolism, and mutations in galactoside 2-alpha-L-fucosyltransferase 2 (FUT2), which catalyzes addition of fucose in α1,2 linkage to Gal at non-reducing ends of glycans, have been linked to susceptibility to IBD.^83^ This has widespread implications for a large subset of the population who are “non-secretors” (i.e., lacking a functional FUT2 enzyme). FUT2 (encoded by the *FUT2* or *Se* locus) controls expression of the blood-group A, B, H(O) antigens in secretory organs including salivary and mucosal tissues, although the extent to which mucus is impacted is unclear.^84^ Non-secretors lack a functional *Se/FUT2* gene (but still have ABO(H) antigen on erythrocytes to due functional FUT1 expression) and thus are missing α1,2 fucosylated glycans on secretions including mucus.^85^ However, whether this loss of function has beneficial or detrimental roles in host–microbiota interactions appears context-dependent. Studies linking *FUT2* mutations and secretor status to IBD susceptibly indicate a significant role in mucosal tolerance to the microbiota. Braun and colleagues have shown this susceptibly is linked to altered microbiota composition and energy metabolism, and increased inflammatory tone of the colon, although mechanisms are unclear.^86^ This may be linked to increased lysophosphatidylcholine production by microbes belonging the genera *Escherichia, Bilophila, Enterorhabdus*, and *Gordonibacter* in the absence of gut epithelial Fut2, which enhanced proinflammatory cytokine production and susceptibility to DSS colitis^87^ (Figure 3A). Sonnenburg and colleagues have shown the influence of fucosylation is dependent on diet, as the microbiota changes induced by fucose deficiency were only apparent in agricultural vs. fiber-free diets.^88^ This important finding may in part explain the conflicting results reported for the impact FUT2 genotype on the human gut microbiota has across different countries^89,90^ since each cohort will likely be associated with different diets. The synthesis of blood-group carbohydrates similarly impacts community structure.^91^

### Role of O-glycan sulfation in tolerance-defense strategies

Besides glycosidic linkages to other sugars, monosaccharides within glycans can be modified further including via sulfation and O-acetylation. In the colon, sulfation is seen on carbon’s 3 and/or 6 of Gal or GlcNAc residues, by the respective Gal-3 or −6 sulfotransferases (STs) or GlcNAc6STs. ^92^ Overall, mucin-type O-glycan sulfation has been associated with tolerogenic functions of mucins (this has been reviewed in Ref. ^18^), although direct roles for its functions on mucins still remains to be resolved. Reduced sulfation of mucins has been associated with UC.^93^ In mice, genetic approaches targeting various proteins in the sulfation pathway, including the enzyme carbohydrate sulfotransferase 4 (CHST4, a.k.a. N-Acetylglucosamine 6-O-sulfotransferase-2 or GlcNAc6ST2), and the Na(+)/Sulfate cotransporter NaS1 (SLC13A1), have shown reducing sulfation impact mucins and is associated with increased susceptibility to colitis^94,95^ (reviewed in ^18^). Nas1 deficiency also increased susceptibly of systemic invasion of the pathogen *Campylobacter jejuni*, showing sulfation limits systemic spread;^95^ this points to a tolerance mechanism of sulfation against *C. jejuni* that limits its invasion, although it is unclear whether mucin sulfation is the sole source for this protection (Figure 3A). Like all glycan modifications, sulfation requires a donor, in this case 3′-phosphoadenosine-5′-phosphosulfate (PAPS), for enzymatic sulfation. Recently, using a conditional genetic strategy to delete the enzyme PAPS Synthase 2 (PAPSS2) in gut epithelium, which promotes donor synthesis, it was demonstrated that sulfation defects in epithelial cells led to enhanced sensitivity to colitis and lumen-to-systemic spread of the colon microbiota,^96^ suggesting a more generalized tolerance strategy of sulfation toward the microbiota. However, PAPS loss in epithelium also affected sulfation of bile acids, so the relative impact of mucin-type O-glycans and other sulfated molecules is still unclear.^96^

**Figure 4.**
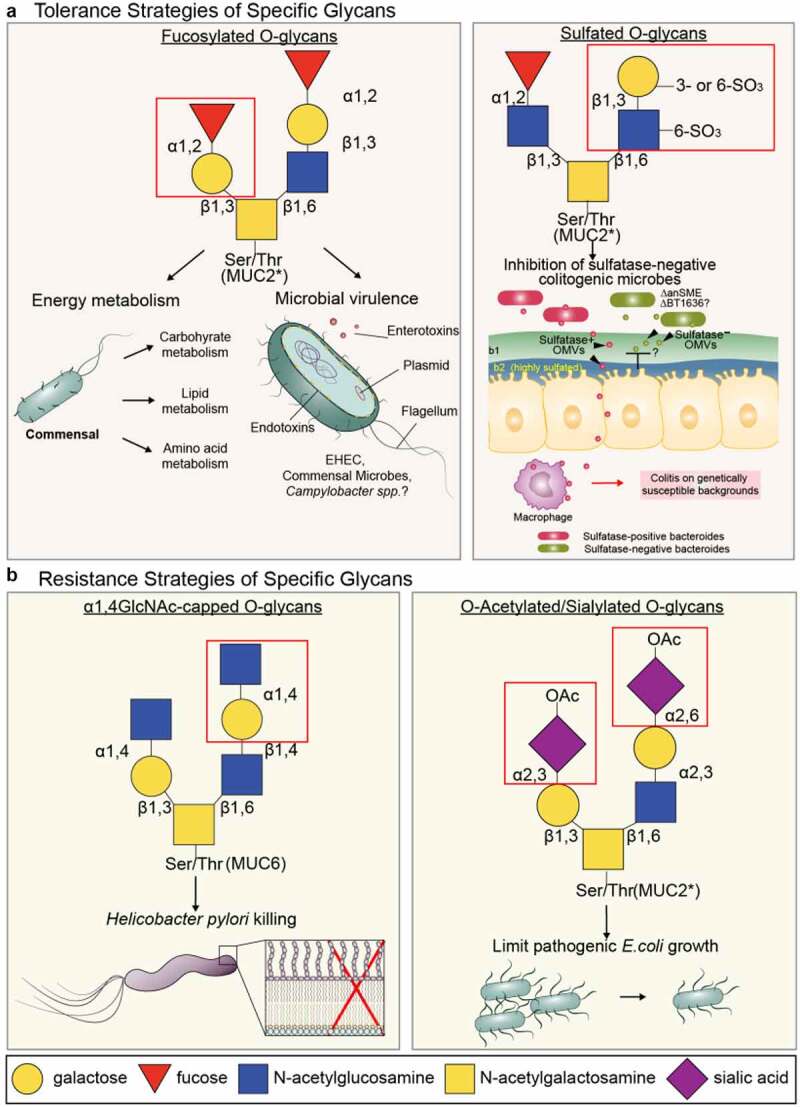
Examples of individual actions of O-glycans promoting host defense strategies of mucus. **A**. tolerance strategies of fucosylated and sulfated O-glycans. fucosylated glycans including O-glycans are associated with a functionally altered microbiota with respect to energy metabolism and virulence in commensal and pathogenic microbes. Sulfation in the intestinal tract occurs on several glycoconjugate classes including mucin-type O-glycans. A direct role in promoting tolerance is supported by studies showing sulfatases expressed in outer membrane vesicles (OMV) of *B. theta* are required to break the mucus and epithelial barrier and activate proinflammatory programs in macrophages on colitis -susceptible genetic backgrounds. Without these sulfatases (anSME), glycan sulfation prevents these colitogenic microbes to causes disease. It is unknown whether the BT1636, the major sulfatase in mucin breakdown, is sufficient to mediate OMV-induced colitis. **B**. Known resistance strategies of specific modifications of O-glycans as illustrated. The killing effect of βα1,4 linked GlcNAc capped O-glycans has been demonstrated from MUC6 from the gastric mucosa (details in text), but not yet from MUC2. The inhibition of pathogenic *E.coli* growth by O-acetylation of sia is due to the inability of *E. coli* to remove the O-acetyl group to access the underlying sia for nutrients (ref. 109).

Sulfatase activities are present in a wide variety of microbiota.^97^ Recent studies have implicated sulfatase activities of major gut symbionts in mucus degradation and colitis. In an elegant, comprehensive glycomics study, Martens and colleagues have used colonic mucins purified from pig distal colons to show a mucus O-glycan-dependent induction of a specific sulfatase (BT1636^3S-^^Gal^) in *Bacteroides thetaiotaomicron* (*B. theta*) that was sufficient to initiate breakdown of complex mucin-type O-glycans for growth and metabolism.^98^ Despite the abundance of sulfatases in *B. theta*, loss of BT1636 alone impaired O-glycan utilization and colonization fitness in vivo.^98^ Whether this translated to a broken-down mucus layer in vivo and disease is unclear, but earlier studies by Stappenbeck and colleagues have shown a direct relationship between *B. theta* sulfatases and colitis. *B. theta* is a potent inducer of colitis on genetically susceptible backgrounds (*CD4-dnTgfb2;IL10rb^−/−^*)^99^ and must employ sulfatases to remove sulfate groups from monosaccharides in order to cause colitis.^100^ This was determined by ablation of the *B. theta* anaerobic sulfatase maturing enzyme (anSME), a post-translational activator of all *B. theta* sulfatases: *B.theta* lacking an SME was not only unable to induce colitis, but consistent with Luis et al.,^98^ had reduced ability to use O-glycans as a nutrient source in vitro.^100^ Importantly, the sulfatase activity was enriched in outer membrane vesicles (OMVs) released from *B. theta*, which presumably breaks through the mucus layer to activate underlying macrophages (Figure 4).^100^ Because the targeting of an SME broadly inhibits sulfatases, it would be informative to learn if this is mediated by BT1636^3S-^^Gal^ identified by Martens and colleagues.^98^ Ultimately, this suggests that mucin sulfation has driven an evolutionary energy investment in the production of sulfatases in *B. theta* to access nutrients and that sulfation of mucus is protective against potentially pathogenic microbes that do not harbor sulfatases (Figure 4A). Our recent work characterizing the newly described MALII^+^ b2 layer produced by distal colon goblet cells shows this layer is highly sulfated and associated with protection from spontaneous and chemically induced colitis.^47^ This was notable since the b1 layer is responsible for most of the barrier functions of the encapsulating mucus layer (Figure 1).^47^ Further work will need to be done to define how this sulfation pattern influences tolerance functions of the mucus.

Sulfation also regulates the functional biology of *B. fragilis*, a gut symbiont with potent anti-inflammatory activities^101^ and positive influences on neurobiology.^102^ By examining mice monocolonized with *B. fragilis* and examining their responses within the lumen, mucus, and tissues of the proximal colon using an innovative hybrid selection RNA sequencing (hsRNA-Seq) approach, Mazmanian and colleagues have shown crude native mucin from the proximal colon specifically induced a distinct group of CAZymes, including a sulfatase (BF3086) and a glycosylhydrolase (GH; BF3134).^103^ Interestingly, *B. fragilis* mutants lacking either BF3086 or BF3134 were unable to use mucins as a food source in vitro suggesting sulfate liberation, as it is for *B. theta*, is important for *B. fragilis* growth.^103^ While they were still able to occupy the colon to similar numbers as WT stains, sulfatase activities were essential for securing occupation of their niche in vivo when challenged by an invading strain.^103^ Despite this, loss of BF3086 sulfatase activities did not impair the known anti-inflammatory abilities of *B. fragilis* polysaccharide A^101^ in the acute 2,4,6-trinitrobenzene sulfonic acid (TNBS) colitis model, although mutants lacking the GH BF3134 did lead to worsened TNBS-induced mucosal damage associated with reduction of IL10-producing Foxp3^+^ T_regs_.^103^ Because a crude mucus fraction was used to show the induction of the sulfatase and GH activities in this study, it is unclear whether these microbial phenotypes were directly caused by the mucus or its glycans, or how this operates in presence of a complex microbiota. However, these studies show mucus-rich regions of the proximal colon can influence anti-inflammatory activities of *B. fragilis* in the distal colon, consistent with the general tolerance role of proximal colon O-glycans protecting from microbial-induced distal disease.^47^

### Role of Sia O-acetylation in tolerance-defense strategies

O-acetylation is a common modification of Sia residues on carbons 4, 7, 8, and 9.^104^ The 9-O-acetyltransferase, Casd1 (Capsule synthesis 1 domain-containing protein 1) is important for the addition of 9-OAc modifications to Sia in humans and mice.^105^ These OAc groups can spontaneously migrate between carbons 7–9 leaving the C9 open for re-O-acetylation and generation of multi-acetylated forms (e.g., tri-acetylated 7,8,9 OAc Sia).^106,107^ 4-OAc is not common in humans.^104^ Detecting the O-acetyl Sia variants in situ has relied upon histochemical approaches, and more recently exploitation of viral proteins that specifically target specific O-acetyl modifications.^108^ Relatively little is known of the biologic functions of O-acetyl groups in the colon. The microbiota, including viruses, do express sialate-O-acetyl-esterases to remove this modification.^65^ *B. theta* uses the 9-O-acetylesterase EstA to remove 9-OAc to allow pathogenic *E. coli* strains to use their sialidases to liberate Sia for nutritional sources.^109^ Interestingly, the EstA was unable to act on 7-OAc linkages.^109^ Although in vivo studies need to confirm the relevance to bacterial pathogenesis, these studies highlight the complexity of host microbe interactions and reveal the potential importance of Sia-OAc modifications in limiting control of pathogen growth (Figure 4B). Since Sia can protect protein glycoconjugates from proteolytic attack,^110^ O-acetylation likely has an indirect role to maintain stability of glycoproteins including Muc2 from proteases by keeping Sia bound to the glycan, but this remains to be tested.

### O-glycosylation-dependent immune homeostasis driving tolerance towards the microbiota

#### Anti-inflammatory metabolite production

O-glycans are emerging as an important source for microbial-derived metabolites. In the last decade, our understanding of the-metabolome-metabolites derived from the gut microbiota-has grown from seeing this as a metabolic by-product of microbial fermentation to a physiologic force influencing both gut mucosal and systemic physiology including neural function and behavior.^111,112^ It is best described for the gut, where metabolic outputs of glycolytic fermentation in the anaerobic environment leads to production of short-chain fatty acids (SCFA) including propionic, butyric, and acetic acids.^111^ These molecules are important for mutualistic symbiosis by i) promoting anaerobiosis (via depletion of epithelial O_2_ during SCFA oxidation) which drives hypoxia-inducible factor-1α -dependent intestinal barrier functions,^113^^,^^114^ and antagonizes potentially pathogenic Enterobacteraciae representatives (e.g. *Salmonella* spp.);^115^ and ii) promoting an immunosuppressive environment by inducing synthesis of Foxp3 to drive T_reg_ differentiation and subsequent IL-10 production.^111^^,^^116^ Butyrate can also induce human MUC2 expression in vitro,^117^^,^^118^although this phenomenon may be cell line-dependent^119^ and challenging to reproduce in therapeutic trials for human UC patients.^120^ While dietary glycans are an important source for microbial fermentation, recent studies have demonstrated mucin-derived O-glycans as an underestimated and important contributor to these anti-inflammatory metabolites (Figure 2A). A Japanese study of 40 healthy people, 49 UC patients, and 44 CD patients showed the butyrate was reduced in UC and CD patients vs. healthy controls regardless of disease activities with the butyrate levels correlated with *Faecalibacterium prausnitzii* in CD patients.^121^ Interestingly, SCFA production was correlated with mucinase activities rather than O-glycan levels, suggesting a lack of mucinase-dependent n-butyrate-producing activities in the UC colon.^121^ Whether these reflect MUC2-derived glycans is unclear as the study did not verify purification or quality of the fecal mucins. Importantly, in mice fed diets containing commercial porcine gastric mucin (PGM), increased SCFA production correlated with increased production of FoxP3^+^ T_regs_ and IgA^+^ B cells pointing to its anti-inflammatory potential; however, the impact on inflammation was not considered.^121^ Similar studies using commercial PGM showed increased proportion of potential mucin degraders *Hungatella hathewayi, Allobaculum stercoricanis, Clostridium oroticum, Marvinbryantia formatexigens, Lactobacillus johnsonii, Lactobacillus reuteri*, and *Desulfovibrio desulfuricanss*, although their contribution to butyrate production is not well defined.^122^ However, this study identified that among the sugars, GlcNAc was the potent monosaccharide fermented for subsequent n-butyrate production, which correlated with higher T_regs_ and IgA^+^ cells as well as lower inflammatory cytokines in the rat cecum.^122^ Critically, it remains unclear the precise contribution of endogenously produced O-glycans to SCFA. We have shown that by knockout of gut O-glycans in the colon, we disrupt metabolic networks which were associated with inflammation.^47^ However, the absolute concentration of SCFA in the absence of colonic mucin-type O-glycans is still unclear. Aside from SCFA, mucins may indirectly contribute to other tolerogenic metabolites: An informative functional bioinformatic study by Wloardska et al. (2017)^123^ showed that *Peptostreptococcus russellii* thrived on mucins, which promoted its colonization and subsequent metabolism of tryptophan to produce the metabolite indoleacrylic acid which reduced injury caused by DSS.^123^ Whether O-glycans influence such activities remains to be studied.

#### O-glycans and immune homeostasis

Another mechanism of tolerance-promoting actions of mucin-type O-glycans is direct interactions with mucosal immune cells. Cerutti and colleagues in a major study revealed that small-intestinal Muc2 could coat gut bacteria and use its glycans to bind Galectin(Gal)-3.^124^ Gal3 then complexes with Dectin 1 and the FcγRIIB receptor on lumen-sampling lamina propria dendritic cells. This Muc2-receptor complex on dendritic cells (DCs) promoted immunologic tolerance to luminal antigens by activating β-catenin, which suppressed LPS-induced DC-intrinsic nuclear factor κβ-dependent pro-inflammatory cytokine expression but left anti-inflammatory cytokines -also induced by Muc2-receptor complex signaling-intact.^124^ (Figure 2B) Whether this is also true of the colon is not known. Further, a recent study has shown Muc2 limits the clonal deletion of developing lymphocytes to an oral antigen,^125^ likely through its barrier effects to limit antigen exposure. By extension, this could enhance recognition of the microbiota, although how this would impact microbiota tolerance and balance the anti-inflammatory functions is unclear. Interestingly, MUC2 glycosylation has shown to enhance IL-8 production by dendritic cells and neutrophil recruitment in vitro, ^126^ which points to a possible proinflammatory role that may compete with immunoregulatory functions; however, this MUC2 was derived from the colorectal cancer-line LS174T,^126^ so whether this effect is due to a cancer-associated carbohydrate-specific activity, and its role *in vivo*, remains to be determined.

It is notable that Newberry and colleagues have identified a novel and highly counterintuitive function of goblet cells in directing antigen passage of luminal contents directly through its theca to underlying professional antigen-presenting cells.^127^ These “goblet cell-associated antigen passages (GAPs)” are functional at baseline in the small intestine, which direct luminal antigens to immune tolerance-promoting dendritic cells.^127^ In colon goblet cells, GAPs are regulated via innate signaling, where Myd88-dependent signals in goblet cells restrict GAP formation in response to either acetylcholine and the microbiota.^128^ Ultimately, TLR-dependent signaling within goblet cells is a mechanism to limit antigen translocation via GAPs and tone down microbiota-dependent inflammatory environments.^128^ Although goblet cells are specialized for glycosylation and mucus production, it is unclear if any of these features play roles in GAP formation. Due to the propensity of inflammation in Muc2 and O-glycan-deficient mice, it could be predicted that GAP formation may be inhibited, but this immunosurveillance role would be overwhelmed by activation of TLR-independent pathways in neighboring epithelial cells including via inflammasomes.^63^

### O-glycan-dependent modulation of virulence as a tolerance strategy

Aside from food sources, host glycans are emerging as key-signaling molecules recognized by microbes that influence their physiology and virulence. Hooper and Gordon have established the paradigm for this, showing *B. theta* can shape its nutrient environment in a competitive system-via L-fucose sensing and signal transduction.^76^ In the absence of L-fucose, the *B.theta* FucR repressor simultaneously binds to the promoter of the *fucRIAK* operon to inhibit expression of fucose-metabolizing enzymes, while inducing expression at the *CSP* (control of signal production) locus, which regulates expression of the secreted factor that induces α1,2 fucosylated glycoconjugates small intestinal epithelium. In the presence of L-fucose, the FucR repression of *fucRIAK* is lifted to allow fucose metabolism, but the CSP locus is repressed since fucose is now abundant. Thus, FucR coordinately regulates fucose metabolism with fucose generation based on sensing of L-fucose ability.^76^ Glycan sensing abilities go well beyond fucose, as *B. theta* can sense when dietary glycans are missing, ultimately switching its gene expression program to harvest host-glycans as an alternate nutrient source.^129^

Regarding overt pathogens, mucins and their glycans have been shown to modulate virulence of diverse groups of bacterial pathogens. *Campylobacter jejuni*, causative agent of food-borne intestinal infection, is a prototype for mucin-dependent modulation of virulence.^130^ *C. jejuni* interacts with intestinal mucus in chickens, its major reservoir in nature where it exists as a commensal. *C. jejuni* also associates with human MUC2; however, this may actually promote virulence.^131,132^ This species-specific difference in symbiosis with *C. jejuni* likely has to do with the different mucus properties between chickens and humans: Chicken mucus, likely via its O-glycans, can directly attenuate virulence of *C. jejuni* by impairing binding and internalization in vitro,^133^ whereas this MUC2 from human sources appears to enhance virulence by directly upregulating key cytotoxins (e.g., cytolethal distending toxin, flagellin, and mucin-degrading enzymes).^131^ Although limited in its saccrolytic abilities, *C. jejuni* may use L-fucose, perhaps liberated from other commensals, as a nutrient source to enhance growth.^134^ These studies show intestinal mucus has the ability to promote tolerance rather than resistance by limiting virulence, but is dependent upon the biochemical properties and source of the mucus.^133^ More studies are needed to clarify how mucus composition shapes *C. jejuni* virulence and how this relates to host-responses to this important pathogen. *C. jejuni* murine models may facilitate this investigation.^135^

Other examples of mucin-dependent microbial modulation have been revealed. Speradino and colleagues have shown sensing of mucin-derived fucose by pathogens including Enterohemorrhagic *E. coli* (EHEC) 0157:H7 by the two component system (TCS) downregulates *locus of enterocyte effacement* (LEE) expression, which reduced expression of key virulence factors including the Type III Secretion System at regions away from the tissues, presumably to shunt intracellular energy allocation to growth pathways to compete with commensals.^136^ However, this was reversed when EHEC contacted the colon tissue;^136^ therefore, it is difficult to determine whether this is ultimately a tolerance strategy or an example of pathogen subversion of host-glycans to promote infection. Recently, Ribbeck and colleagues have shown O-glycans specifically can impact virulence strategies of other clinically important mucosal pathogens. These include *Pseudomonas aeruginosa*, by reducing biofilm production.^137,138^ Further, mucin O-glycans directly signal through a TCS system to downregulate Type VI secretion system expression and overall virulence of *P. aeruginosa*.^138^ More recently, O-glycans on salivary mucin-derived Muc5B had a similar virulence-suppressing role in the oral pathogen *Streptococcus mutans*.^139^ O-glycans were recognized by *S. mutans*, which downregulated quorum-sensing genes and its ability to acquire antibiotic resistance by impacting expression of competence genes.^139^ Importantly, these studies showed O-glycans did not directly impact microbial burdens. Collectively, these studies highlight a bonafide role of O-glycans in promoting host defense, not by resistance mechanisms that promote clearance, but rather a tolerance mechanism that tunes pathogenic symbiosis toward commensalism. However, as many of these studies were done in vitro, it will be important to demonstrate whether this is recapitulated in vivo. In this regard, how O-glycans dictate similar responses to the commensal microbiota is still unclear. One important study has linked innate lymphoid cell (ILC)-3-dependent IL-22 production to Fut2 expression and fucosylation, which reduced virulence-related gene expression of the microbiota, providing evidence of glycan-dependent microbiota tolerance^140^ (Figure 4A), but the influence of the mucus system in this context remains to be verified.

### Roles of mucus and their glycans in resistance mechanism

#### Direct antimicrobial functions of mucin-type O-glycans

Beyond tolerance functions, mucus can have direct antimicrobial activities, although evidence for this is still limited. One major study shows that α1,4-linked GlcNAc, a unique capping structure on O-glycans of the gel-forming mucin MUC6, can directly promote *H. pylori* killing by inhibiting cell wall synthesis.^141^ Interestingly, this was found on MUC6 deep in gastric glands, and not MUC5AC on the surface mucus cells, and thus was postulated to prevent *H. pylori* invasion^141^ (Figure 4B). Whether similar structures exist within the colon has not yet been shown, nor whether other colonic mucin-type O-glycans directly promote direct antimicrobial activities. It is notable the *C. rodentium* burdens in *Muc2^−/−^* mice are greater vs. WT mice,^38^ raising the possibility that mucins may potentially have antimicrobial activities.

#### Indirect antimicrobial functions of mucin-type O-glycans

Mucins can also serve as indirect mediators of antimicrobial functions. Small intestinal Muc2 serves as a reservoir for antimicrobial peptides, such as alpha-Defensins −1, −2, and −6 and Ubiquicidin among others, which have direct killing capacities against known pathogens including *Bacillus megaterium, Shigella flexneri, Enterococcus faecalis, E. coli*, and *Salmonella Typhimurium*^142^ (Figure 3). These are likely from Paneth cells and neighboring epithelial cells. Both murine and human colon Muc2 have shown similar activities, with human MUC2 exhibiting a diverse array of antimicrobial molecules,^143^ and murine colon Muc2 able to bind beta defensin 2,^144^ the Gram positive-targeting lectin-like ZG16,^145^ flagellated microbiota-targeting LyPD8,^146^ possibly Gram-negative targeting RELMβ,^147^ and a host of other proteins as identified by proteomics.^148^ How O-glycans influence these interactions is unclear; however, studies by Chadee and colleagues have pointed to a more complex dynamic between MUC2 and antimicrobial peptides, with MUC2 stimulating expression of hBD2 while paradoxically protecting enteropathogens like EPEC from the killing capacity of hBD2 in vitro.^144^ These inducing and protecting effects were dependent upon its intact MUC2 O-glycans, but independent of Sia and N-glycan-derived mannose.^144^ Further, mucin synergized with butyrate to induce expression of the cathelicidins LL37 in human mucin producing cell lines via MAP-kinase and cyclic AMP signaling, and the related mCramp in mice, which was enhanced by inflammatory stimuli.^149^ Whether O-glycans are important for this role, or modulate cathelicidin or other antimicrobial peptide killing capacities is unclear, but given that mucin-type O-glycans can be source from microbial-derived butyrate (mentioned above), this may point to a dual role of O-glycans to regulate anti-inflammatory and antimicrobial programs at the interface in part via butyrate production. The findings are summarized in Table 1. Moving forward, it will be important to understand the contribution of the b1- and b2-derived mucus layers in the positioning, expression, and functioning of antimicrobial peptides and other molecules in the intestinal tract.

**Table 1.** Summary of various roles of O-glycans in promoting host defense along the GI tract

Host Defense Strategy	Function	General Mechanism	Glycan Type	Mucin^a^	Disease Protected from	Model	Affected Microbe(s)	Reference
*Tolerance*	Spatial regulation	Stabilization of mucus barrier	O-glycans (core 1- and 3 -derived)	Muc2	Idiopathic microbiota-dependent colitis	Mouse	*Consortium*	48,54,59
*Tolerance*	SCFA production	Immunosuppression via T_reg_ induction	O-glycans (core 3-based)	MUC2^b^	Altered Homeostasis	Human	*Consortium*	121
				Muc2^b^		Rat	*Consortium*	122
*Tolerance*	Immune- regulation	Dendric cell modulation by Muc2-coated microbes	O-glycans	Muc2^b^	Allergy	Mouse	*Consortium*	124
*Tolerance*	Microbial signaling	Regulation of virulence	O-glycans (core 1- and 3 -derived)	Muc5AC/B	Infection	In vitro	*Pseudomonas* *aeroginosa*	138,139
				Muc5B (Salivary)	Infection	In vitro	*Streptococcus mutans*	139
				Muc2	Intestinal infection	In vitro	*C.jejuni*	132,133
*Tolerance*	Barrier function	Preventing systemic spread	Mucin sulfation ^c^	Muc2^b^	Infection	In vivo	*C.jejuni*	95
		Mucus barrier function			Colitis and colon cancer		*Consortium*	96
				Muc2	Colitis		*Sulfatase-negative B. thetaiotaomicron*	100
*Tolerance*	Microbial modulation	Selective colonization	2,3 linked Milk Sialyllactose	N/A	Colitis	In vivo	*Consortium*	150
*Tolerance*		Induction of CAZymes in symbionts	O-glycans	Muc2^b^	Colitis	In vivo	*B.fragilis*	103
*Resistance*	Microbicidal	Inhibition of cell wall synthesis	O-glycan (α1,4 GlcNAc)	Muc6	Gastric infection	Ex vivo	*H. pylori*	141
*Resistance*		Indirect via mucus stabilization which allows sequestration of antimicrobial peptides	Unknown	Muc2 (small intestine)	Intestinal infection	Ex-vivo	*S. Typhimurium* *S.flexneri*, *E.faecalis* *E. coli*, *Bacillus megaterium*	142
*Resistance*				MUC2		Ex-vivo	*E.faecalis* *E. coli*, *S.aureus*. *C. albicans*	143
*Resistance ^d^*		Co-operation with Butyrate or IL-β1β to stimulate antimicrobial gene expression	Non-sialylated O-glycans (for hBD2)	MUC2 (cell line) Muc2 (murine colon)		In vitro (MUC2), In vivo (Muc2).	*Enteropathogenic E. coli*	144,149
*Resistance ^e^*	Colonization resistance?	Possible induction of CCF genes in commensal that enable saturation of niches otherwise occupied by pathogenic species	LacNAc	Muc2^e^	Intestinal infection	In vivo	*Enterotoxic* *B. fragilis*	55,151

^a^Nomenclature indicates original source of the mucin-type O-glycan (e.g., MUC2, human; Muc2, mouse)

^b^Presumed based on location where mucus was extracted, but not verified in references

^c^Cannot rule out other sulfated biomolecules in this phenotype, e.g., bile acid sulfation

^d^Muc2-Oglycans also prevent killing capacity, pointing to a tolerance effect

^e^Inferred role, but not formally demonstrated

### O-glycans and colonization resistance strategies

Polysaccharide utilization loci (PULs) encode a group of coordinated genes encoding CAZymes, transporters, and sensors that work in concerted fashion to bind, degrade, import, and metabolize extracellular glycans to boost microbial fitness.^20,152^ CAZymes include Glycoside Hydrolayzes (GHs) that liberate sugars via hydrolysis of glycosidic bonds; Glycosyltransferases (GTs) that covalently link monosaccharides via glycosidic bonds; Carbohydrate Esterases (CEs) to remove carbohydrate modifications; Polysaccharide Lyases (PL) that target polysaccharides containing uronic acid, and Carbohydrate Binding Modules within CAZymes that recognize precise glycan motifs.^153^ PULs are based on the paradigm of starch utilization systems (SUS) expressed by *B.theta* that break down starch polymers, and most PULs encode SUS-like systems.^154^ Although many PULs are dedicated to plant-derived glycans, several are also dedicated to host-derived O-glycans.^155^ These systems have been well-characterized and are critical for microbial fitness of several species;^156^ however, studies are also implicating them in host fitness. Recently, Mazmanian and colleagues have identified a novel PUL, encoding a SUS-like system with 5 genes (*ccfA-E*) called *c*ommensal *c*olonization *f*actors (CCF) within *B. fragilis* that is essential for stable colonization of *B. fragilis* within a unique niche within proximal colon crypts, where it protected *B. fragilis* from washout following bacterial infection or antibiotic disturbance.^54^ Although the PUL was induced by N-acetyllactosamine (LacNAc) structures typically found on mucin-type O-glycans, it was unlikely CCF genes utilized these structures to mediate their functions.^54^ Importantly, the CCF system was important for expression of a capsular polysaccharide, PSC, and, indirectly, PSB, which together were essential for stable colonization within mucus-enriched regions of the proximal colonic mucosal surface.^157^ Mechanistically, this was due to CCF-dependent induction of secretory IgA (sIgA), which promoted *B. fragilis* aggregation within colon mucus and ultimately stable colonization amongst a complex microbiota.^157^ The discovery of this system, which was present in other *Bacteroides spp* (*B. vulgatus, B. thetaiotaomicron*)^54^ has implications for pathobionts like enterotoxigenic *B. fragilis* (ETBF), a potent aggravator of colitis and colitis-associated cancer via elaboration of *B.fragilis* toxin (BFT) and activation of Stat3-dependent epithelial proliferation and TH17-dependent inflammation:^158^ Does CCF (via PSB/C) have the capability to prevent the related ETBF from colonizing and inducing disease? One study by Sears and colleagues has supported this notion, showing that non-toxigenic *B. fragilis*, if colonized first, can reduce likelihood of ETBF from colonizing and inducing colitis-associated cancer.^151^ Conversely, ETBF colonization cannot be reversed by addition of non-toxigenic *B. fragilis*. Critically, the protective effect was not dependent upon the anti-inflammatory *B. fragilis* PSA, but rather directly correlated with strain dominance.^151^ This finding is consistent with Lee et al.^54^ suggesting *B. fragilis* and ETBF ultimately occupy the same niche, although whether this is CCF-dependent remains to be determined. Ultimately, these studies point to a possible novel mechanism of O-glycan and mucus-dependent resistance mechanisms whereby O-glycan-related structures activate CCF genes to potentially saturate niches, promoting resistance to colonization by pathogenic variants of the same species. While mucin-type O-glycans are capable of inducing CCF production,^54^ whether this is truly Muc2 O-glycan-dependent in vivo, and whether CCF genes recognize, metabolize, or mediate their effects through mucin-type O-glycans, remains to be determined. Notably, Muc2 can prevent BFT-dependent damage and lethality following ETBF colonization, further underscoring the importance of mucus in the *B. fragilis* activities in the gut.^159^ This was mediated via a TCS that regulated BFT expression; it would be informative to learn if this TCS was modulated by O-glycans on mucus.

### O-glycosylation as a double-edged sword

#### Microbial adhesins, O-glycans, and pathogenesis

While glycosylation overall is essential for intestinal health, O-glycans can also promote detrimental interactions with the microbiota, including pathogens. Many pathogens possess adhesins that bind mucin carbohydrates and influence pathogenesis, with two major outcomes: 1. The adhesin-glycan interaction acts as a decoy to limit recognition of cell surface glycans.^12^ 2. The adhesin-glycan interaction acts as an initial binding site to facilitate infection.^160^ It is not always clear what the precise role of the adhesin-glycan interaction is in host defense without gain- and loss-of-function approaches. For example, it is established that *Helicobacter pylori* binds to histo-blood group antigens H-type-1 structures and Le^b^ antigens via its group antigen-binding adhesin (BabA).^84^ Studies in *Fut2-/-* mice, as well as in humans who are nonsecretors as discussed above (i.e., loss of function alleles in FUT2), reveals the loss Blood Group A on gastric mucins renders tissues refractory to infection by BabA^+^ strains,^161,162^ highlighting a role in pathogenesis. Similarly, inflammation-induced increases in sialyl-Le^a^/^x^ epitopes are subverted by *H. pylori* Sia-binding adhesin (SabA) to promote binding and persistence during chronic inflammation.^163^ *Vibreo cholera* uses its chitin-binding protein (GpBA) to bind to GlcNAc residues in intestinal mucin and induce mucin secretion;^164^ this is a pathologic interaction because loss of GpA, reduced adherence, colonization and signs of pathology in a murine model.^164^ A more thorough review of adhesins, including fimbrial adhesins and flagellin as expressed by pathogenic *E. Coli* strains, is given in Refs.^71,160^ In general, however, adhesions represent subversion mechanisms by pathogens to facilitate colonization, and likely reflect the limits by which the diverse O-glycome can mediate their multifarious protective roles without compromising them for commensal and mutualistic symbionts that can potentially promote colonization resistance. In this regard, commensal strains of *Lactobacilllus* have mucin-binding proteins that are thought to limit its biogeography to the niche layer of mucus.^165^ The carbohydrate binding module 40 of *Ruminococcus gnavus* transialidase RgNanH binds to 2,3/6-linked sialylated glycans on mucus, which is important for its ability to forage mucus.^166^ *B. fragilis* can bind directly intestinal Muc2, which is important for tolerance-promoting attributes including protection from colitis as discussed above. With our evolving understanding of the mucus system, it will be important to understand how mucin binding influences pathogenesis in the context of microbiota encapsulation and its travel down the intestinal tract, as the rate of adhesions and migration through the mucus must be faster than its removal via evacuation. Mouse models of *C. rodentium, S. Typhimurium, C. jejuni*, or ETBF infection would be an ideal system to explore this question.^38,39,135,159^

#### Dr. Jekyll and Mr. Hyde: Context-dependent functions of glycans in microbe-dependent diseases

Whether a glycan or a monosaccharide present in glycans promotes beneficial or maladaptive interactions with the microbiota can be highly context-dependent as demonstrated by in vivo models and studies with milk Oligosaccharides (MOs) which are structurally identical to many mucin-type O-glycans.^82^ For example, current data suggest sialylated glycans can be a double-edged sword: In landmark studies by the Gordon lab, the presence of Sia on human MOs was enough to completely reverse growth defects induced by a dysbiotic microbiota isolated from a malnourished Malawian infant.^167^ However, Sias liberated from *Bacteroides vulgatus* sialidase activities on mouse MOs can drive pro-inflammatory *E. coli* expansion and exacerbate acute inflammation in mice.^168^ α2,3-linked sialyllactose on mouse MOs can also aggravate colitis in *il10^−/−^*mice potentially through TLR4 activation in dendritic cells.^169^ Conversely, the same mouse MO-derived α2,3 sialyllactose can promote development of a less colitogenic microbiota as assessed in DSS colitis.^150^ It is notable that the related α2,6-linked sialyllactose did not have the same protective effect, highlighting how subtle differences in glycan linkages have profound impacts on microbiota-dependent colitis.^150^ While the MOs in these studies are not derived from mucin, these structures are abundant on MUC2, raising questions whether α2,3 linked Sia conjugates in the MUC2 niche layer contribute to disease risk. To this end, liberation of Sia from mucins by commensal bacteria can cause infectious colitis by the inadvertent outgrowth of antibiotic resistant pathogens (*C. difficile*, and *S. Typhimurium*) after antibiotic exposure.^170^

Fucosylation has a similar checkered history: While α1,2 linked fucosylation can reduce risk of IBD, lack of fucosylation may actually be beneficial to prevent *Helicobacter pylori* as discussed above.^84^ Similarly, blood-group A synthesis on mucus glycans promotes *S. Typhimurium* invasion and worsens disease: both features were reduced in mice lacking B4Galnt2, which was comprised of a microbiota that reduced *S. Typhimurium* pathogenicity and which was associated with increased mucus thickness.^91^ Interestingly, the dual nature of O-glycosylation might be played with the same pathogens: In vitro, using human transformed colorectal lines, core 2 O-glycans have been shown to influence EPEC and EHEC O157:H7 infection by enhancing their adherence to,^171^ while also limiting invasion into, the cells.^172^ Collectively, these studies (summarized in Table 2) show O-glycan-dependent tolerance strategies can sometimes be subverted by pathogens to promote infection.

**Table 2.** Summary of various roles of O-glycans and related structures in perpetuating microbe-dependent diseases along the GI tract

Maladaptive (O-)glycan Function	General Mechanism	Glycan Type	Mucin^a^	Relevant Disease	Model	Affected Microbe(s)	Reference
invasion	Modulation of microbiota	Blood group antigens	Muc2	Intestinal infection	Mouse	*S. Typhimurium*	91
promoting growth	Commensal + antibiotic-dependent Sia liberation	Sialylated O-glycans	Muc2^b^	Intestinal infection	Mouse	*C. difficile* *S.Typhimurium*	170
	Sia metabolism by proinflammatory *E. coli*	2,3 linked Sialylated Milk O-ligosaccharides	N/A	Acute colitis	Mouse	*E. coli*	168
	Pathogenic *E. coli* growth	O-acetylated Sialic Acid	N/A	Intestinal infection	In vitro	*E. coli*	109
increase pathogen adherence	Unknown	Core 2-derived O-glycans	MUC2	Intestinal infection	In vitro/ Cell line	*EPEC and EHEC:O157H7*	171,172
	Blood group antigen–binding adhesin (BabA)	βα1,2 Fuc	MUC5AC^b^	Gastric infection	Mouse	*H. pylori*	84
	Sialic acid binding adhesin (SabA)	Sialylated O-glycans	MUC5AC^b^				163
	Promoting virulence	Complex O-glycan	MUC2	Intestinal infection	In vitro	*C.jejuni*	131

^a^Nomenclature indicates original source of the mucin-type O-glycan (e.g., MUC2, human; Muc2, mouse)

^b^Presumed based on intestinal location where mucus was studied, but not verified in reference

## Summary and future studies

As shown with the in vivo models described above, it is clear that mucins and their component glycans give rise to a mucus system of extraordinary structural and chemical complexity (Figure 1), which contribute to the ecosystem in ways that have profound impacts on host physiology and host–microbe interactions. Remarkably, many of these interactions seem to fine-tune microbial and host physiology to favor host tolerance toward commensal and pathogenic microbiota (Figures 2–4). However, mucin-type O-glycans can tip the balance in favor of susceptibility to microbial infections and ensuing tissue damage. This highlights the need to understand the glycome itself and how it specifically changes prior to and during disease states, so as to better understand the primary defects leading to infection and disease. Although this review focused on bacteria, this clearly extends to other microbial classes (i.e., archaea, unicellular, and multicellular eukaryotic parasites – e.g., fungi, amoebae, and nematodes, viruses, and prions). Here, capitalizing on in vivo models of glycan deficiencies will be essential for this goal, as well as confirming the significance of related in vitro studies. Using such models, it will be important to dissect the relative contributions of the newly defined b1 and b2 layers to resistance and tolerance strategies as they represent distinct Muc2 subtypes with unique glycomes. Further, we need to understand how the glycosylation of mucins is regulated, which will identify how we can intervene to modify the glycome to maximize host-microbial mutualism and an adaptive symbiosis, including with pathogens (e.g., mimicking the mucus-dependent commensalism with *C. jejuni* as observed in chickens). To this end, a focus should be on the glycosyltransferases that mediate the biosynthesis of O-glycans, or understanding how the microbiota itself can tailor the O-glycome. This will allow context-specific and tunable glycosylation in vivo to maximize mutualistic interactions with our resident microbes.

## Figures and Tables

**Table:** 

Abbreviation	Full Name
Gal	Galactose
GlcNAc	N-acetylglucosamine
GalNAc	N-acetylgalactosamine
Fuc	Fucose
Sia	Sialic Acid
9-OAc-Sia	9-O-acetyl-sialic acid
Sd^a^/Cad	Sid antigen
Le ^a/b/x/y^	Lewis-a,b,x or y antigen
ST6Gal1	Beta-galactoside alpha-2,6-sialyltransferase 1
C1galt1	Core 1 beta1,3 galactosyltransferase (core 1 synthase)
C3GnT	Core 3 beta1,3 N-acetylglucosaminyltransferase (core 3 synthase)
GT	Glycosyltransferase
GH	Glycoside Hydrolase
CasD1	N-acetylneuraminate 9-O-acetyltransferase
CHST4	N-Acetylglucosamine 6-O-sulfotransferase-2 or Carbohydrate sulfotransferase 4
FUT2	Galactoside 2-alpha-L-fucosyltransferase 2
ST6GalNAc1	Alpha-N-acetylgalactosaminide alpha-2,6-sialyltransferase 1
Muc2	Mucin-2
PGM	Porcine Gastric Mucin
MALII	*Maackia amurensis* agglutinin lectin (2,3Sia-or sulfate binding)
UEA1	Ulex europaeus agglutinin (a1,2 Fucose-binding)
OMVs	Outer membrane Vessicle
HPLC	High Performance Liquid Chromatography
LC/MS	Liquid Chromatography mass spectometry
MO	Milk Oligosaccharide
anSME	anaerobic sulfatase maturing enzyme
PAPS	3′-phosphoadenosine-5′-phosphosulfate
ETBF	Enterotoxigenic *B. fragilis*
BFT	*B. fragilis* toxin
CCF	Commensal Colonization Factor
TCS	Two component system
PUL	Polysaccharide Utilization Loci
SUS	Starch Utilization System
CAZyme	Carbohydrate Active Enzyme
SCFA	Short-chain fatty acid
Treg	Regulatory T cell
Myd88	Myeloid Differentiation Factor- 88
IBD	Inflammatory Bowel Disease
UC	Ulcerative Colitis
CD	Crohn’s Disease
GAP	Goblet cell-associated antigenic passages
